# Supplementary material for: Cryo-electron tomography of cardiac myofibrils reveals a 3D lattice spring within the Z-discs
Source: Commun Biol. 2020 Oct 16;3:585. doi: 10.1038/s42003-020-01321-5 (PMC7567829; doi:10.1038/s42003-020-01321-5)
Supplement: Supplementary file 1 — Supplementary Information [file 42003_2020_1321_MOESM1_ESM.pdf]

Supplementary Information for:

Cryo-electron tomography of cardiac myofibrils reveals a 3D lattice spring within the Z-discs

Authors: Toshiyuki Oda, and Haruaki Yanagisawa.

Supplementary Figures 1 – 3
Supplementary Movies 1 – 5

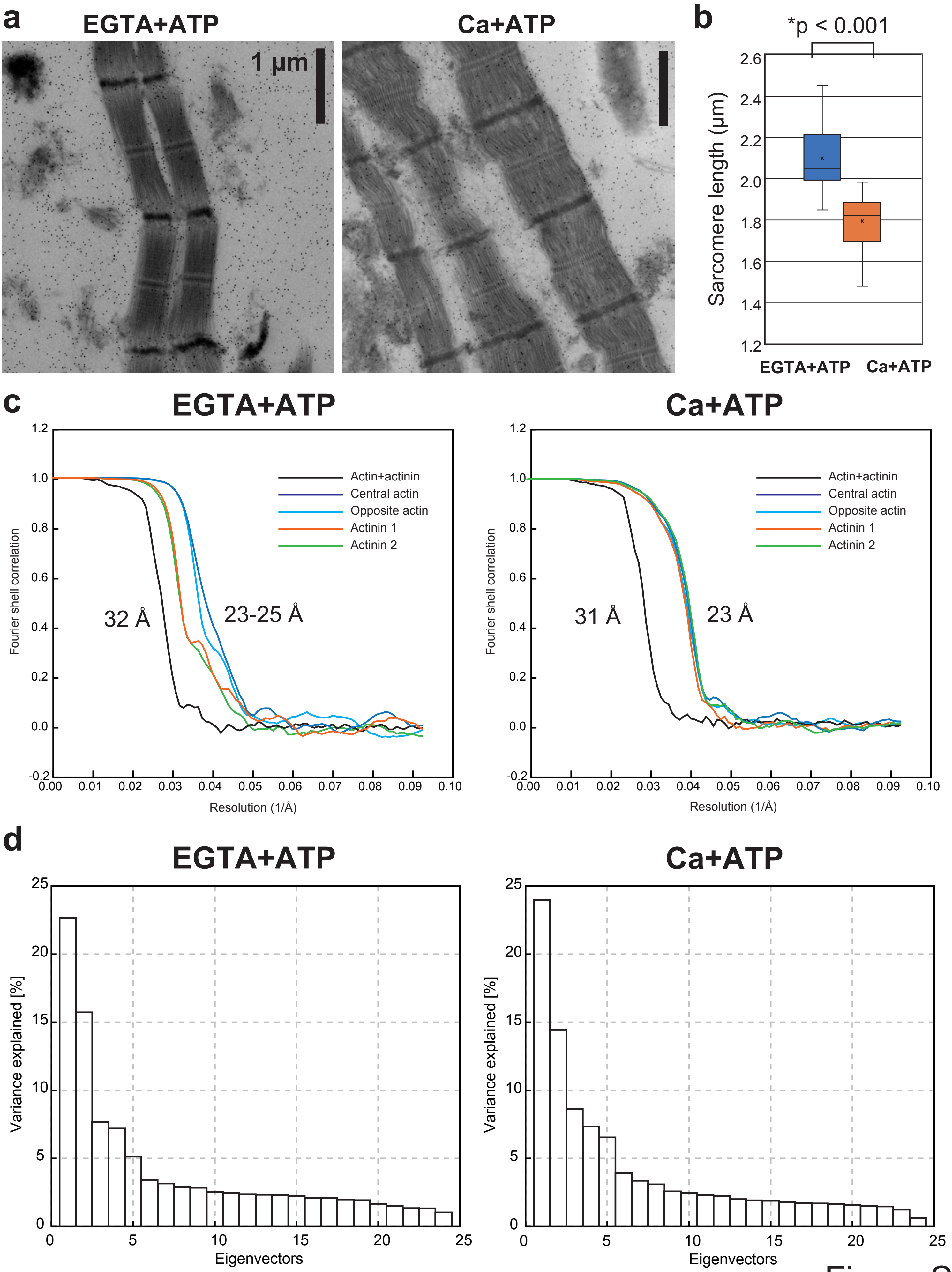
a
EGTA+ATP
Ca+ATP
1 µm
b
*p < 0.001
Sarcomere length (µm)
2.6
2.4
2.2
2.0
1.8
1.6
1.4
1.2
EGTA+ATP
Ca+ATP
c
EGTA+ATP
Ca+ATP
Fourier shell correlation
Resolution (1/Å)
Actin+actinin
Central actin
Opposite actin
Actinin 1
Actinin 2
32 Å
23-25 Å
31 Å
23 Å
d
EGTA+ATP
Ca+ATP
Variance explained [%]
Eigenvectors

Figure S1

Supplementary figure 1

(a) Related to Fig. 1. Longitudinal sections of myofibrils observed by ultra-thin section electron microscopy. Note that we used cross-sections of myofibrils for thin section tomography in Fig.1. (b) Related to Fig. 2. Comparison of the sarcomere lengths between the two ionic states. Sarcomere lengths (Mean ± SD, n = 25 for each state) were determined by measuring the distances between the midpoints of the two neighboring Z-discs. The observed length change was similar to the previous study [18]. The asterisk indicates statistical significance (p=8.0×10^{-10}, t=7.4, one-sided, Students' t-test). (c) Related to Fig. 4a. Fourier shell correlation plots calculated using Relion-3 post-processing. Resolutions of the averaged tomograms were limited to 31-32 Å (black line, actin+actinin). Multibody refinements improved the resolutions up to 23-25 Å. Opposite actin: the opposite-polarity actin; actinin 1: the α-actinin molecules in orange that bind to the central F-actin; actinin 2: the α-actinin molecules in green that bind to the opposite-polarity F-actin. (d) Related to Fig. 4b. Contributions of the eigenvectors to the variance. The first and the second eigenvectors explained nearly 40% of the variance in both states.

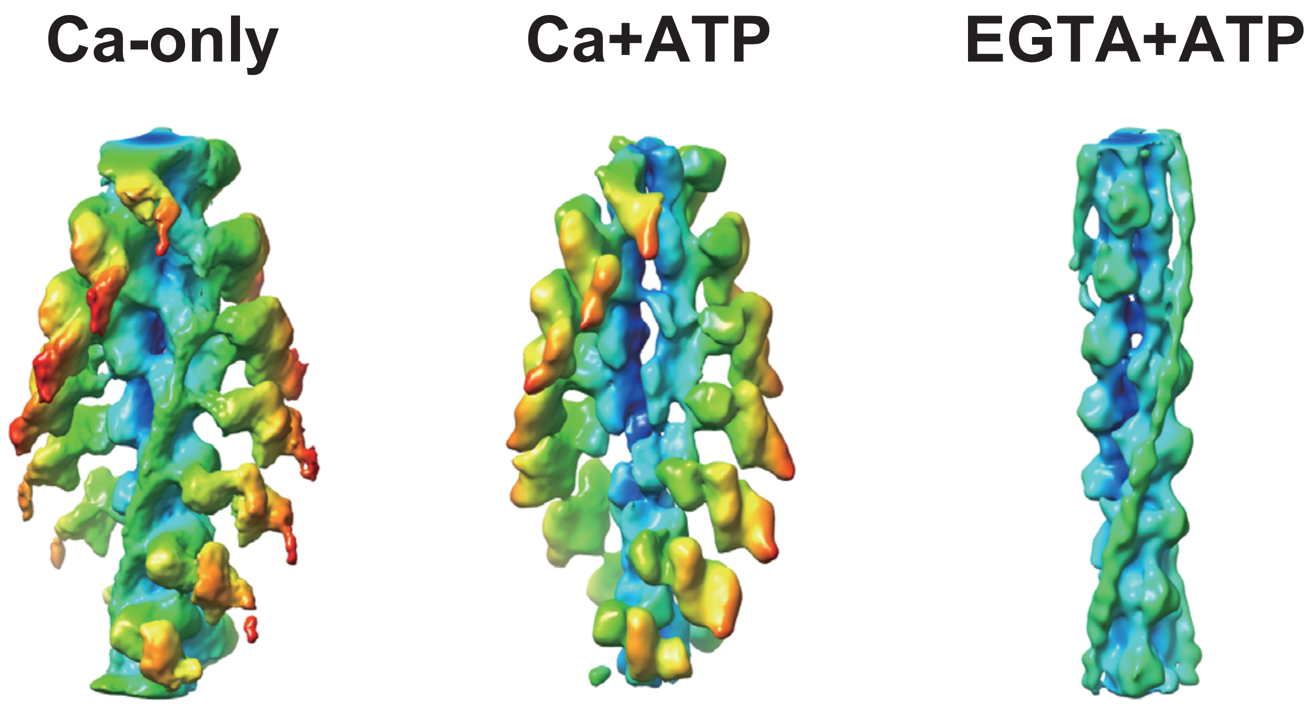

Supplementary Figure 2

Supplementary Figure 2

Related to Fig. 2. Averaged subtomograms of the thin filament in the A-band in Ca-only, Ca+ATP, and EGTA+ATP states. In Ca-only state, ATP was omitted from the buffer. The thin filaments were manually picked from the tomograms and subtomograms were averaged using PEET and Relion Refine3D. The numbers of subtomograms averaged were as follows: Ca-only, 3,476; Ca+ATP, 5808; and EGTA+ATP, 3309. No symmetry was imposed and the periodicity of troponin was ignored. The maps were colored according to the distance from the long axis of the filament. Both in Ca-only and Ca+ATP states, the thin filaments were decorated with the myosin heads, which were dissociated in EGTA+ATP state. In the Ca+ATP state, the thin filament region was not clearly visualized, especially the tropomyosin densities were unclear, probably due to the structural heterogeneity between the actively-moving myosin heads and the thin filament.

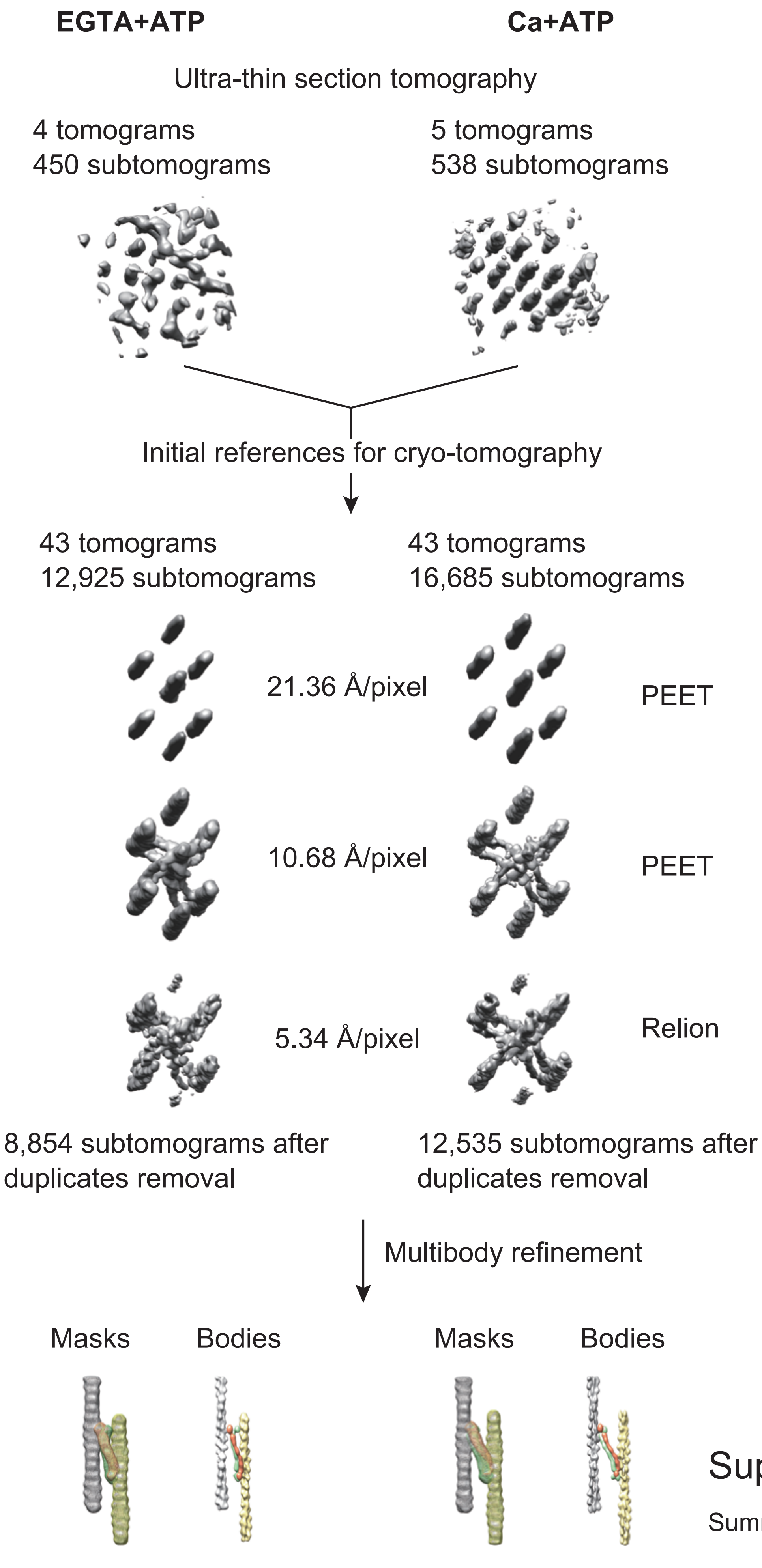

Supplementary Figure 3

Summary of the reconstruction scheme.
