# Supplementary material for: Cryo-electron tomography of cardiac myofibrils reveals a 3D lattice spring within the Z-discs
Source: Commun Biol. 2020 Oct 16;3:585. doi: 10.1038/s42003-020-01321-5 (PMC7567829; doi:10.1038/s42003-020-01321-5)
Supplement: Supplementary file 3 — Description of Additional Supplementary Files [file 42003_2020_1321_MOESM3_ESM.pdf]

Description of Additional Supplementary Files

Title: Supplementary Movie 1

Description: Related to Fig. 3a. Averaged subtomograms in the EGTA+ATP and the Ca+ATP states were shown.

Title: Supplementary movie 2

Description: Movie representation of Fig. 4a. Composite maps in the EGTA+ATP and the Ca+ATP states, composed of one central F-actin (gray), four opposite-polarity F-actins (yellow), sixteen α-actinin monomers (orange and green). The -actinin crystal structures (PDB 4D1E) were fitted into the α -actinin maps (red and green models).

Title: Supplementary movie 3

Description: Related to Fig. 4b. Movie representations of the first and the second eigenvectors. Gray, Central F-actin; yellow, opposite-polarity F-actin; orange, α-actinin dimer.

Title: Supplementary movie 4

Description: Related to Fig. 5. Morphing movies showing a trajectory between the F-actin-actinin models in EGTA+ATP and Ca+ATP states. Gray: F-actin; green and orange: α-actinin.

Title: Supplementary movie 5

Description: Related to Fig. 5. Morphing movies showing a trajectory between the α-actinin models in EGTA+ATP and Ca+ATP states. Green and orange: α-actinin.
